# Non-ideal gas behavior matters in hydrodynamic instability

**DOI:** 10.1038/s41598-022-26629-6

**Published:** 2022-12-21

**Authors:** Jie Ren, Markus Kloker

**Affiliations:** 1 Institute of Aerodynamics and Gas Dynamics, University of Stuttgart, Pfaffenwaldring 21, 70569 Stuttgart, Germany; 2 Beijing Institute of Technology, 100081 Beijing, People’s Republic of China

**Keywords:** Aerospace engineering, Mechanical engineering, Fluid dynamics

## Abstract

Hydrodynamic instability, the foundation for flow’s laminar-turbulent transition and various predicting models, has been helping to understand the physics and shape the design of aerodynamic devices. While for hypersonic flow it is clear that thermodynamic/-chemical effects need be accounted for due to the high temperatures occurring, this letter unveils that also for low-speed flow at ambient temperatures non-ideal, i.e. real-gas effects can play a strong role—a feature missed by the classic theory for Newtonian fluids. By considering a three-dimensional low-speed boundary-layer flow in different thermodynamic regimes—subcritical, supercritical and transcritical—we show the importance of coupling thermodynamics by sensitivity studies of the perturbation growth rate to various inputs of the full stability equations. High sensitivities are found, and not only the transition-onset location but also the transition mechanism may be concerned.

## Introduction

When studying flow instability it is broadly acknowledged that a flow with an inflectional velocity profile generally bears inviscid instability whose growth rate is strong, paving the way towards expeditious laminar-turbulent transition. The theoretical support dates back to Rayleigh’s inviscid theory^1^, and Fjørtoft’s improved criterion^2^. Both assume inviscid perturbations in the stability equation (inviscid Orr-Sommerfeld equation) and allow the baseflows to be viscous but incompressible. The inflection-point criterion, although serving formally only as a necessary condition for inviscid instability, has led to a substantial and intuitive understanding simply by looking at the base-flow velocity profiles, constituting the cornerstone of classic monographs on hydrodynamic stability^3,4^. The success of the inflection-point criterion extends to the perception of the mechanisms of various controlling strategies, e.g., pressure gradient, wall suction & blowing, and wall heating & cooling from the alteration of the base-flow velocity profiles^5^. Specifically, the general non-dimensional boundary-layer equation for a 2D baseflow reads, derived from the streamwise momentum for a fluid with non-constant viscosity and density:

1 $$\begin{aligned} \begin{aligned} \frac{\partial ^{2}u}{\partial y^{2}}=&\frac{Re}{\mu }\rho u\frac{\partial u}{\partial x}+\underset{\text {gradient}}{\underset{\text {pressure}}{\underbrace{\frac{Re}{\mu }\frac{dp}{dx}}}}+\underset{\text {blowing}}{\underset{\text {suction}/}{\underbrace{\frac{Re}{\mu } \rho v\frac{\partial u}{\partial y}}}}-\underset{\text {cooling}}{\underset{\text {heating}/}{\underbrace{\frac{1}{\mu }\frac{\partial \mu }{\partial y}\frac{\partial u}{\partial y}}}}. \end{aligned} \end{aligned}$$

In this article, *u*, *v*, *w* are used for streamwise (*x*), wall-normal (*y*) and spanwise (*z*) velocity components. *p* and $\mu$ denote pressure and dynamic viscosity. All variables have been non-dimensionalized with fixed reference values and for the pressure $(\rho u^2)_{\text {ref}}$ is used. *Re* is the fixed Reynolds number based on the reference values, and $\mu$ and $\rho$ are identical to one if there is no alteration of the viscosity and density with respect to their reference values which is the standard case for incompressible flow. Subscript *w* is used for wall parameters. Using (1), one concludes that for a standard Blasius (flat-plate) boundary layer (no pressure gradient, controlling nor heat transfer), ${\partial ^{2}u}/{\partial y^{2}}|_w=0$, indicating that the inflection point falls strictly on the wall; hence inviscid instability is absent. Further it is concluded that, upon controlling, a favorable pressure gradient ($dp/dx<0$), wall suction ($v_w<0$), or cooling wall for ideal gases ($\partial \mu /\partial y|_w>0$) shift the inflection point inside the wall and shape the velocity profiles fuller. The flow is consequently linearly more stable (and vice versa). When friction is included in the stability consideration the viscous Orr–Sommerfeld (O–S) equation results (For given baseflow profiles *u*(*y*) and *w*(*y*), at given *x* and *Re* one solves for the spatial growth rate $\alpha _i$ and streamwise wavenumber $\alpha _r$ of a fixed-frequency disturbance growing exponentially in *x*-direction, see the description of Eqs. (5) and (6). Viscous instability adds, does not need an inflection point in the velocity profiles, is much weaker than inviscid instability, and the above findings equally hold. Though widely used by researchers and engineers, we shall note that the O–S framework essentially misses physics in the aspect of thermodynamics brought by the fluid itself. Therefore, the completeness and truthfulness of the conventional hydrodynamic stability theory remains unclear.

The present work investigates Newtonian fluids , i.e. fluids where the viscosity does not depend on the shear in the flow but on temperature, density or pressure of the fluid. We note in passing that non-Newtonian, viscoelastic flow instabilities^6^ amount to an own subject that witnesses essential applications in daily life and industry (e.g. blood flow, healthcare products, engineering inks). One of the most striking attributes is that the Reynolds number supporting the instabilities can be significantly lower than in Newtonian flows. A shred of earlier evidence for this is the work by Forbes^7^, who proposed that even weakly non-Newtonian effects can give rise to instabilities.

**Figure 1:**
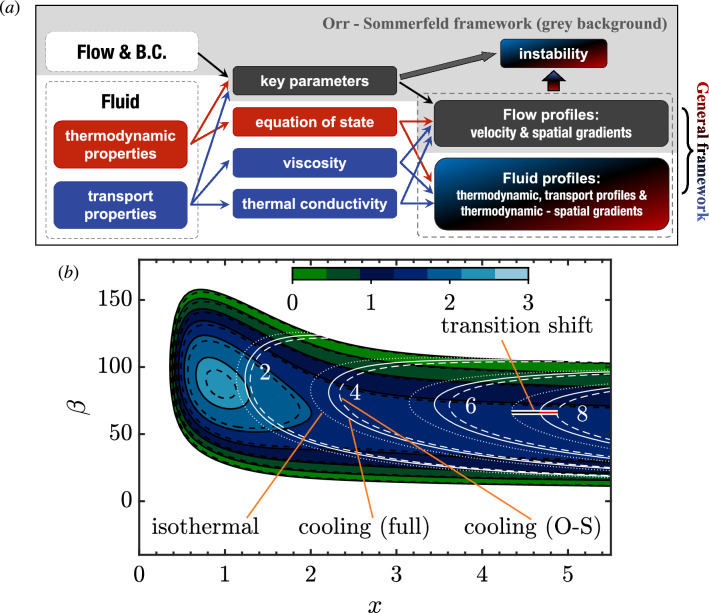
(**a**) An overview of essential inputs in building a general flow stability analysis framework. The grey background encircles the classic O–S framework, where only the Reynolds number and velocity profiles bear the physics. (**b**) Stability diagram for the steady cross-flow mode in the $x-\beta$ frame. Spatial growth rates $-\alpha _i$ (black lines) and N factors (white lines) are compared for the full (solid lines) and O–S (dashed lines) equations.

## Results

### Overview of the general framework for flow instability with non-ideal fluids

Figure 1a encapsulates the constituent inputs toward the linear stability analysis of flows. The problem is defined both by the flow and the running fluid. In the conventional O–S framework, fluids are assumed to be incompressible, ideal, and isothermal such that the energy equation is dropped, and the physical role of the fluid thus gets minimized. Interested readers are referred to Eq. (6) of "Methods" section. Assuming incompressible flows and a perfect gas, the classical stability theory solves a simplified eigenvalue problem. In a general framework, in addition to the velocity profiles, the state-variable relation (equation of state) & transport profiles (viscosity and thermal conductivity), as well as their thermodynamic gradients, become essential inputs in the stability problem. Moreover, the velocity profiles are altered by the fluids’ properties (as the classical framework accounts for). All the key parameters, e.g., Reynolds, Mach, Prandtl, and Eckert numbers, correspondingly depend on fluid properties. Carry this general view with Eq. (1), the involvement of pressure gradient and wall suction & blowing in (1) stays within the O–S framework. However, the explanation of the effect of wall cooling/heating using the inflection-point criterion is ill-defined. The reason is that wall cooling/heating not only influences the velocity profile but also calls for integrating the intrinsic physics of the fluid, especially in the stability equations. As an example we consider the stability of a three-dimensional boundary-layer flow because this is the most representative for the flow on rotating blades of energy conversion machines^8^. Two fundamentally different instabilities can occur—cross-flow (CF) and Tollmien–Schlichting-type (TS) instability. An alteration in the primary stability properties can not only lead to a transition-location shift but also to a different transition mechanism and thus control measure^9^. The baseflow corresponds to an (ideal-air) experiment^10,11^ and mimics a low-speed swept flow over a wing surface subject to a favorable pressure gradient, where CF modes (comprehensive reviews are available through^12,13^) are responsible for the laminar-turbulent transition. Researchers and engineers rely on the integral of growth rates—the N factor—in determining the transition position^14^. Figure 1b compares the growth rates and N factors of steady CF modes between the full stability equations for an ideal gas (solid lines) and the standard O–S setting with constant density and viscosity (dashed lines). The N factor amounts to the integral of the spatial growth rate starting from the neutral position of a considered disturbance:

2 $$\begin{aligned} {N=\mathop {{\mathop {\intop }\nolimits _{\left. x\right| _{\alpha _{i}=0}}^{x}}}\limits (-\alpha _{i})\text {d}x; \; -\alpha _i=\frac{\text {d}}{\text {d}x}\ln (A), \text { where } A \text { is the amplitude of the perturbation. } } \end{aligned}$$

The flow is subject to wall cooling with $T_w/T_\infty =15/16$. We show the isolines of N-factors with values of 2, 4, 6, and 8, where the uncontrolled isothermal reference case (dotted lines) is included to indicate the effect of wall cooling. As can be seen, if a wind tunnel measured $N=8$ to indicate transition onset, the isothermal case has a transition position $x_\text{iso}=4.34$. Considering wall cooling, the full equations give the transition shift $\Delta x_\text{full} = 0.33$ while the classical O–S setting predicts $\Delta x_\mathrm{O-S} = 0.52$ which is 58.8% more than the correct transition shift. Using the classical O–S framework thus considerably overestimates the impact of wall cooling because the thermodynamic & transport property effects in the stability equations partly compensate the effect of the altered base-flow profiles.

As an essential step in building a more physical system, Lees and Lin^15^ derived the generalized-inflection-point criterion for compressible flows, incorporating the contribution of flow temperature. Mack^16^ advanced the viscous theory for compressible flows. He found a change in the mathematical nature of the stability equations when the Mach number increases, leading to the well-known Mack’s higher modes (often dominate flow transition in hypersonic flows). However, in the compressible scheme, the physics of the fluid largely stayed in the ideal gas regime due to the erstwhile aerodynamic demand. On the other hand, the O–S framework has been extended to account for temperature-dependent viscosity^17,18^, coupling with various controlling methods^19^. In line with the emphasis on viscosity, viscosity-stratified flows amount to a particular type that attracted significant attention^20^. For example, their instabilities have been revealed for flows with horizontal heat advection^21^, mixing layer of miscible fluids^22,23^. Along different paths towards turbulence^24^, viscosity stratification plays a minimal role in algebraic instability^25^. Contrarily, the edge state is considerably modified by viscosity gradients leading to a change of threshold for transition^26^. Seen from Fig. 1a, viscosity is only one part of the real fluid physics^27,28^. An other is the state-variable relation, provoking the effect of non-ideal fluid properties, also called real-gas effects, if the pressure is high and intermolecular forces have a direct impact on the fluid behavior. Will the stability framework with ideal-gas assumption, provided with the non-ideal base-flow profiles, give the right prediction? We aim to answer this question through this article, which shall call for attention to integrate thermodynamics into hydrodynamic stability theory and contribute to a better understanding of flows with substantive fluids, e.g., blood flows^29^, geophysical flows^30,31^, superfluid turbulence^32^ and dense flows^33^.

**Figure 2:**
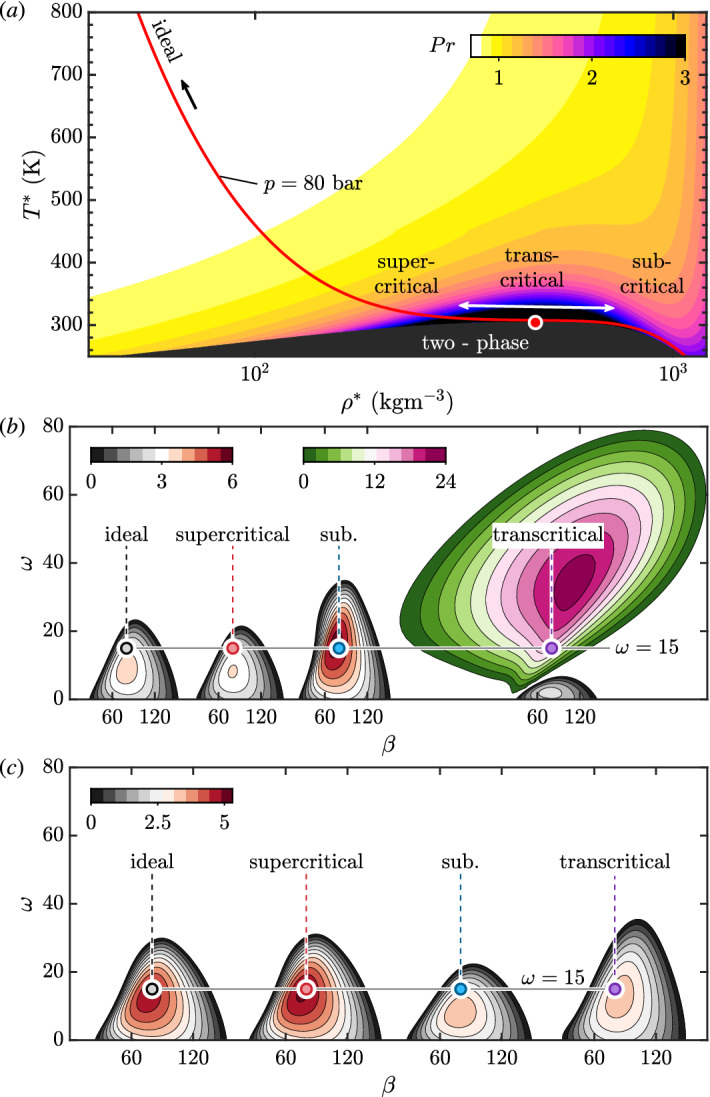
(**a**) Temperature–density diagram of CO2 colored by the Prandtl number. The solid red line corresponds to the isobar of 80, near which a three-dimensional boundary layer is considered. The red dot marks the critical point close to the Widom line; the ideal, supercritical, transcritical, and subcritical regimes can be seen. (**b**, **c**) Stability diagrams for different regimes with (**b**) wall cooling and (**c**) heating in the $\beta -\omega$ frame. In panel (**b**), we have used a separate color map for the inviscid Tollmien–Schlichting mode (outmost right), whose growth rate is significantly larger. The colored dots point to modal instability with $\beta =80$ and $\omega =15$ discussed in Fig. 3.

### Three-dimensional boundary-layer instability of supercritical CO_2_

Without loss of generality we consider supercritical fluids near the critical point. These fluids are recognized as important working mediums in the energy sector^8^, while their highly non-ideal thermodynamic & transport properties challenge the conventional framework. Figure 2a shows the temperature–density ($T-\rho$) diagram of CO_2_ colored by Prandtl number ($Pr=\mu C_p/\kappa$). $C_p$ and $\kappa$ stand for isobaric specific heat capacity and thermal conductivity, respectively. We will focus on the isobar of 80 along which strong non-ideality occurs on crossing the pseudo-critical point (coinciding with the critical point at the critical pressure), also known as the Widom line^34^ that characterizes the physics of pseudo-boiling, defined as the isobaric maximum of $C_p$. The ideal regime reported in Fig. 1a corresponds to the high-temperature regime (above 600 K). As can be seen, the Prandtl number only behaves as a constant in the ideal regime while showing strong variations near the critical point, highly dependent on the thermodynamic state. With this fluid and a look-up table for its thermodynamic properties^35^, we obtain the three-dimensional boundary-layer stability diagram in the $\omega -\beta$ frame for $x=1$ in Fig. 2b and c for wall cooling and heating, respectively. A clear comparison indicates that the effect of wall cooling/heating is highly dependent on the fluid’s thermodynamic regime. For example, the flow supports another mode with tremendous growth rates and instability band in the transcritical regime. This mode is of inviscid Tollmien–Schlichting(/Kelvin–Helmholtz) type, i.e. with the wave phase speed about oriented in main-flow direction for highest amplification, which may be expected with adverse but not at all with favorable pressure gradient^9^. Instead of CF-type transition, with involvement of steady CF vortices originating from minute surface roughness, a TS-type flow transition will set in here, despite the favorable pressure gradient. The dramatic difference suggests that new physics is present when integrating thermodynamics. Besides, the effect of wall heating gets strengthened in the supercritical regimes but less pronounced in the sub- and transcritical regimes. What has caused such large differences, the baseflow velocity or other items shown in Fig. 1a?

**Figure 3:**
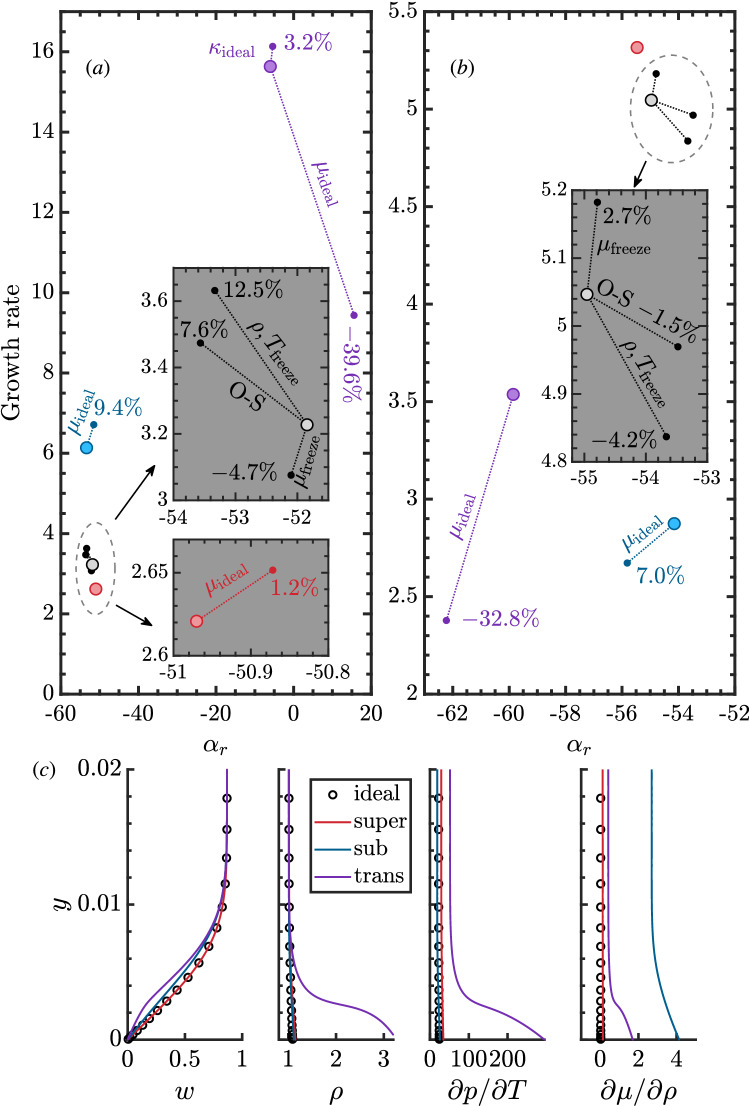
Discrete spectrum of the linear stability corresponding to the colored dots in Fig. 2b and c, $\beta =80,~\omega =15$. Panel (**a**) is for Fig. 2b, cooling wall, and panel (**b**) for Fig. 2c, heating wall. We consider the influence of freezing certain inputs and O–S equations in the ideal regime. The results are denoted with small solid circles with the error of growth rate labeled. In the three non-ideal regimes (red, blue, magenta), we present the influence using ideal viscosity and thermal conductivity inputs. (**c**) Distribution of *w*, $\rho$, $\partial p/\partial T$, and $\partial \mu /\partial \rho$ as functions of *y* for the wall-cooling case. They belong to the velocity and density profiles and thermodynamic gradients originating from the state-variable relation and the transport properties (see Fig. 1a).

**Figure 4:**
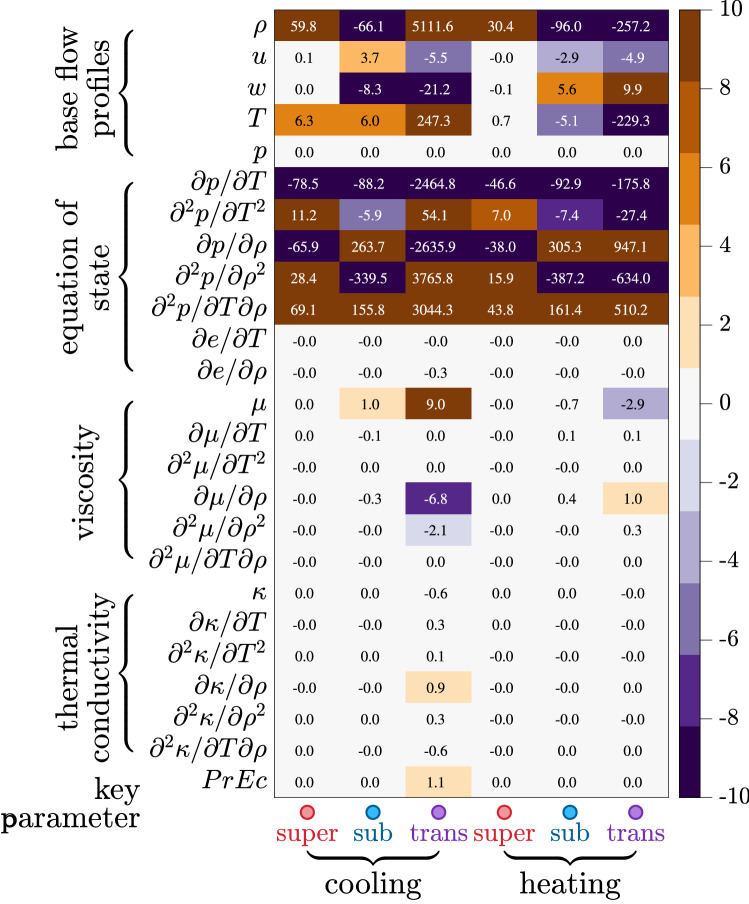
Sensitivity $\epsilon$ of the spatial growth rate to different inputs of the stability operator. These inputs correspond to different categories shown in Fig. 1a. The six columns are for supercritical, subcritical, and transcritical regimes with wall cooling and heating conditions, respectively (see bottom of the figure).

### The role of non-ideality

Figure 3a and b compares a representative modal state from the stability diagram, $\beta =80$ and $\omega =15$, for wall cooling and heating respectively. The spatial growth rate $-\alpha _i$ and wavenumber $\alpha _r$ are displayed. The color style is matched to Fig. 2b and c and throughout the article, indicating different thermodynamic regimes. A zoom-in of the ideal regime indicates how different simplifications bias the results. For example, the O–S framework leads to a growth rate error of 7.6%/− 1.5% for wall cooling/heating. Solely freezing viscosity or density & temperature profiles respectively also leads to noticeable eigenvalue changes, showing that even with an ideal gas, an accurate N-factor can not be obtained with a simplified framework once a temperature gradient exists. Considering that a conventional stability framework takes empirical laws for transport properties, we show that it is inadequate to use this framework with the non-ideal baseflow as input. In particular, the growth rate demonstrates significant errors by replacing the viscosity ($\mu$ and related gradients) with ideal laws (e.g., Sutherland laws). We shall also note that the influence of thermal conductivity ($\kappa$ and related gradients) is much smaller and only appears crucial in the transcritical regime with cooling.

Besides transport properties, Fig. 3c compares the base-flow profiles for *w*, $\rho$, $\partial p/\partial T$, $\partial \mu /\partial \rho$. Noticeably, the velocity profiles (*w*(*y*)) alter in different thermodynamic regimes. In particular, the transcritical case with wall cooling shows an inflection point alongside strong density alteration near the wall, causing inviscid TS instability. To understand a fluid’s thermodynamic role comprehensively, we present the sensitivity $\epsilon$ of the growth rates to each input of the stability equations, defined as

3 $$\begin{aligned} \varepsilon =\left. \frac{\partial \alpha }{\partial \sigma }\right| _{\sigma \rightarrow 0},\;\chi =\sigma \chi _{\text{ideal}}+\left( 1-\sigma \right) \chi _{\text{nonideal}} \end{aligned}$$

Here $\chi$ stands for any physical input, e.g., *T* and $\partial \mu /\partial \rho$. Thus the sensitivity measures how the stability depends on the non-ideal inputs relative to conventional ideal assumptions. Figure 4 shows a summary of each term at different thermodynamic regimes. A first conclusion is drawn for all the non-ideal regimes: The velocity and thermodynamic profiles & gradients exert the most critical influence (top 10 rows of the table except the pressure), whereas the transport properties, as discussed with Fig. 3a and b, are less influential, albeit they already show a significant influence (viscosity related terms). Therefore it is essential to account for all the thermodynamical physics of the fluid (including transport properties) to obtain base profiles correctly (e.g., $\rho$, *u*, *T*, $\partial p/\partial T$). Examing Fig. 4 column by column, one concludes that the transcritical regime bears the highest sensitivities. This is due to its most substantial non-ideality on crossing the Widom line. It is also noteworthy that terms like $\partial \mu /\partial \rho$ and $\partial \kappa /\partial \rho$ are usually ignored in conventional studies. Therefore even with correct base-flow profiles, the hydrodynamic stability framework must be upgraded to incorporate all the related profiles unless the ideal-gas assumption is met in a practical situation.

## Discussion

Our study highlights the importance of accurately including the thermodynamic properties of the (non-ideal) fluid missed in conventional hydrodynamic stability theory when a temperature gradient in the investigated shear layer exists. More generally, when a fluid is operated near its critical point such that highly non-ideal thermodynamic effects are present, the conventional framework fails to give the correct growth rate, dominating mode, and consequently, prediction of the transition physics, which may render a predesigned control measure pointless. A sensitivity study shows that thermodynamics, viz. the state-variable relation (equation of state) and the viscosity (including its thermodynamic gradients), but not the thermal conductivity, must be adequately accounted for in the base-flow profiles *and* hydrodynamic stability framework. This also means that using an approximate, analytical equation of state like the Van-der-Waals model or other^27^ may palpably deteriorate the stability outcome.

## Methods

### The laminar baseflow

We solve the ‘parabolised’ Navier–Stokes equations (PNS) for the laminar baseflow. In steady boundary-layer flows without separation, the streamwise viscous gradient is much smaller than the other component in wall-normal direction. The PNS are thus derived from the complete Navier–Stokes equations by dropping the streamwise gradient in the viscous terms^36^.

We employ subscript *e* for boundary-layer edge values. Here $\rho _{e}\left( x\right)$ and $T_{e}\left( x\right)$ are potential/irrotational-flow values given by the isentropic relations:

4 $$\begin{aligned} S\left( \rho _{e}\left( x\right) ,p\left( x\right) \right) =S\left( \rho _{\infty },p_{\infty }\right) ,\;S\left( T_{e}\left( x\right) ,p\left( x\right) \right) =S\left( T_{\infty },p_{\infty }\right) , \end{aligned}$$

where *S* stands for entropy, and *p*(*x*) is the measured pressure with $dp/dx<0$. The PNS are integrated downstream using an implicit Euler scheme, starting from an initial profile at $x=x_0$. In this study, we prescribe the stream- and spanwise velocities $u(x_0,y)$, $w(x_0,y)$ using the Falkner–Skan–Cooke (FSC) solution and $v(x_0,y)=0$. The thermodynamic variables ($\rho , T$) are given either as the potential-flow values (applying isentropic relations) or extrapolated from existing downstream data. The validation of the PNS results with different initial profiles was made, ensuring that the influence of the initial profiles is insignificant.

### Linear stability with fully included thermodynamics

We consider linear modal instability of the laminar baseflow. The theory was derived based on the state postulate that a simple compressible system is defined by two independent thermodynamics properties. The perturbation vector is defined as ${\varvec{q}}=\left( \rho ^{\prime },u^{\prime },v^{\prime },w^{\prime },T^{\prime }\right) ^{T}$. Therefore, perturbations in the other transport and thermodynamic properties (e.g., $e^{\prime }$, $\kappa ^{\prime }$) are formulated as a function of $(\rho ^{\prime },T^{\prime })$ through two-dimensional Taylor expansion^37^. The stability equations are obtained from the N–S equations by subtracting the governing equations of the unperturbed baseflow, formulated as

5 $$\begin{aligned} {\varvec{\mathsf{{ L}}}}_{\varvec{\mathsf{{ t}}}}\frac{\partial {\varvec{q}}}{\partial t}+{\varvec{\mathsf{{ L}}}}_{\varvec{\mathsf{{ x}}}}\frac{\partial {\varvec{q}}}{\partial x}+{\varvec{\mathsf{{ L}}}}_{\varvec{\mathsf{{ y}}}}\frac{\partial {\varvec{q}}}{\partial y}+{\varvec{\mathsf{{ L}}}}_{\varvec{\mathsf{{ z}}}}\frac{\partial {\varvec{q}}}{\partial z}+{\varvec{\mathsf{{ L}}}}_{\varvec{\mathsf{{ q}}}}{\varvec{q}} +{\varvec{\mathsf{{ V}}}}_{{\varvec{\mathsf{{ xx}}}}}\frac{\partial ^{2}{\varvec{q}}}{\partial x^{2}}+{\varvec{\mathsf{{ V}}}}_{{\varvec{\mathsf{{ xy}}}}}\frac{\partial ^{2}{\varvec{q}}}{\partial x\partial y}+{\varvec{\mathsf{{ V}}}}_{{\varvec{\mathsf{{ xz}}}}}\frac{\partial ^{2}{\varvec{q}}}{\partial x\partial z}+{\varvec{\mathsf{{ V}}}}_{{\varvec{\mathsf{{ yy}}}}}\frac{\partial ^{2}{\varvec{q}}}{\partial y^{2}}+{\varvec{\mathsf{{ V}}}}_{{\varvec{\mathsf{{ yz}}}}}\frac{\partial ^{2}{\varvec{q}}}{\partial y\partial z}+{\varvec{\mathsf{{ V}}}}_{{\varvec{\mathsf{{ zz}}}}}\frac{\partial ^{2}{\varvec{q}}}{\partial z^{2}}=0. \end{aligned}$$

The definition of the matrices in Eq. (5) is provided in ref^27^. The normal-mode form, ${\varvec{q}}\left( x,y,z,t\right) =\hat{{\varvec{q}}}\left( y\right) \exp \left( i\alpha x+i\beta z-i\omega t\right) +c.c.$ is used, and we seek the solution in the spatial mode, where the spanwise wavenumber $\beta$ and angular frequency $\omega$ are given as real input and $\alpha$ is the complex eigenvalue to be determined by the resulting nonlinear eigenvalue problem:

6 $$\begin{aligned} {\varvec{\mathsf{{ L}}}}\hat{{\varvec{q}}} = 0. \end{aligned}$$

Here ${\varvec{\mathsf{{ L}}}}$ is a 5-by-5 matrix. Its detailed expressions are given as:

7 $$\begin{aligned}{} & {} {\varvec{\mathsf{{ L}}}}_{1,1}=-i\alpha u+i\omega -i\beta w,\;{\varvec{\mathsf{{ L}}}}_{1,2}=-i\alpha \rho ,\;{\varvec{\mathsf{{ L}}}}_{1,3}=-\rho D-\frac{\partial \rho }{\partial y},\;{\varvec{\mathsf{{ L}}}}_{1,4}=-i\beta \rho ,\;{\varvec{\mathsf{{ L}}}}_{1,5}=0 \end{aligned}$$

8 $$\begin{aligned}{} & {} {\varvec{\mathsf{{ L}}}}_{2,1}=-i\alpha \frac{\partial p}{\partial \rho }+\frac{1}{Re}\frac{\partial \mu }{\partial \rho }\frac{\partial u}{\partial y}D+\frac{1}{Re}\frac{\partial \mu }{\partial \rho }\frac{\partial ^{2}u}{\partial y^{2}}+\frac{1}{Re}\frac{\partial u}{\partial y}\left( \frac{\partial ^{2}\mu }{\partial \rho ^{2}}\frac{\partial \rho }{\partial y}+\frac{\partial ^{2}\mu }{\partial \rho \partial T}\frac{\partial T}{\partial y}\right) \end{aligned}$$

9 $$\begin{aligned}{} & {} {\varvec{\mathsf{{ L}}}}_{2,2}=-\frac{\alpha ^{2}\left( 2\mu +\lambda \right) }{Re}-i\alpha \rho u+i\omega \rho +\frac{1}{Re}\frac{\partial \mu }{\partial y}D-i\beta \rho w+\frac{\mu }{Re}D^{2}-\frac{\beta ^{2}\mu }{Re}\end{aligned}$$

10 $$\begin{aligned}{} & {} {\varvec{\mathsf{{ L}}}}_{2,3}=\frac{i\alpha \left( \mu +\lambda \right) D}{Re}+\frac{i\alpha }{Re}\frac{\partial \mu }{\partial y}-\rho \frac{\partial u}{\partial y},\;{\varvec{\mathsf{{ L}}}}_{2,4}=-\frac{\alpha \beta \left( \mu +\lambda \right) }{Re}\end{aligned}$$

11 $$\begin{aligned}{} & {} {\varvec{\mathsf{{ L}}}}_{2,5}=-i\alpha \frac{\partial p}{\partial T}+\frac{1}{Re}\frac{\partial \mu }{\partial T}\frac{\partial u}{\partial y}D+\frac{1}{Re}\frac{\partial \mu }{\partial T}\frac{\partial ^{2}u}{\partial y^{2}}+\frac{1}{Re}\frac{\partial u}{\partial y}\left( \frac{\partial ^{2}\mu }{\partial T^{2}}\frac{\partial T}{\partial y}+\frac{\partial ^{2}\mu }{\partial T\partial \rho }\frac{\partial \rho }{\partial y}\right) \end{aligned}$$

12 $$\begin{aligned}{} & {} {\varvec{\mathsf{{ L}}}}_{3,1}=\frac{i\alpha }{Re}\frac{\partial \mu }{\partial \rho }\frac{\partial u}{\partial y}-\frac{\partial p}{\partial \rho }D+i\beta \frac{1}{Re}\frac{\partial \mu }{\partial \rho }\frac{\partial w}{\partial y}-\frac{\partial ^{2}p}{\partial \rho ^{2}}\frac{\partial \rho }{\partial y}-\frac{\partial ^{2}p}{\partial \rho \partial T}\frac{\partial T}{\partial y}\end{aligned}$$

13 $$\begin{aligned}{} & {} {\varvec{\mathsf{{ L}}}}_{3,2}=\frac{i\alpha \left( \mu +\lambda \right) D}{Re}+\frac{i\alpha }{Re}\frac{\partial \lambda }{\partial y},\;{\varvec{\mathsf{{ L}}}}_{3,4}=i\beta \frac{1}{Re}\frac{\partial \lambda }{\partial y}+\frac{i\beta \left( \mu +\lambda \right) D}{Re}\end{aligned}$$

14 $$\begin{aligned}{} & {} {\varvec{\mathsf{{ L}}}}_{3,3}=-\frac{\alpha ^{2}\mu }{Re}-i\alpha \rho u+i\omega \rho +\frac{1}{Re}\frac{\partial \left( 2\mu +\lambda \right) }{\partial y}D-i\beta \rho w+\frac{2\mu +\lambda }{Re}D^{2}-\frac{\beta ^{2}\mu }{Re}\end{aligned}$$

15 $$\begin{aligned}{} & {} {\varvec{\mathsf{{ L}}}}_{3,5}=\frac{i\alpha }{Re}\frac{\partial \mu }{\partial T}\frac{\partial u}{\partial y}-\frac{\partial p}{\partial T}D+i\beta \frac{1}{Re}\frac{\partial \mu }{\partial T}\frac{\partial w}{\partial y}-\frac{\partial ^{2}p}{\partial T^{2}}\frac{\partial T}{\partial y}-\frac{\partial ^{2}p}{\partial \rho \partial T}\frac{\partial \rho }{\partial y}\end{aligned}$$

16 $$\begin{aligned}{} & {} {\varvec{\mathsf{{ L}}}}_{4,1}=\frac{1}{Re}\frac{\partial \mu }{\partial \rho }\frac{\partial w}{\partial y}D-i\beta \frac{\partial p}{\partial \rho }+\frac{1}{Re}\frac{\partial \mu }{\partial \rho }\frac{\partial ^{2}w}{\partial y^{2}}+\frac{1}{Re}\frac{\partial w}{\partial y}\left( \frac{\partial ^{2}\mu }{\partial \rho ^{2}}\frac{\partial \rho }{\partial y}+\frac{\partial ^{2}\mu }{\partial \rho \partial T}\frac{\partial T}{\partial y}\right) \end{aligned}$$

17 $$\begin{aligned}{} & {} {\varvec{\mathsf{{ L}}}}_{4,2}=-\frac{\alpha \beta \left( \mu +\lambda \right) }{Re},\;{\varvec{\mathsf{{ L}}}}_{4,3}=i\beta \frac{1}{Re}\frac{\partial \mu }{\partial y}+\frac{i\beta \left( \mu +\lambda \right) D}{Re}-\rho \frac{\partial w}{\partial y}\end{aligned}$$

18 $$\begin{aligned}{} & {} {\varvec{\mathsf{{ L}}}}_{4,4}=-\frac{\alpha ^{2}\mu }{Re}-i\alpha \rho u+i\omega \rho +\frac{1}{Re}\frac{\partial \mu }{\partial y}D-i\beta \rho w+\frac{\mu }{Re}D^{2}-\frac{\beta ^{2}\left( 2\mu +\lambda \right) }{Re}\end{aligned}$$

19 $$\begin{aligned}{} & {} {\varvec{\mathsf{{ L}}}}_{4,5}=\frac{1}{Re}\frac{\partial \mu }{\partial T}\frac{\partial w}{\partial y}D-i\beta \frac{\partial p}{\partial T}+\frac{1}{Re}\frac{\partial \mu }{\partial T}\frac{\partial ^{2}w}{\partial y^{2}}+\frac{1}{Re}\frac{\partial w}{\partial y}\left( \frac{\partial ^{2}\mu }{\partial T^{2}}\frac{\partial T}{\partial y}+\frac{\partial ^{2}\mu }{\partial T\partial \rho }\frac{\partial \rho }{\partial y}\right) \end{aligned}$$

20 $$\begin{aligned}{} {} {\varvec{\mathsf{{ L}}}}_{5,1}&= -i\alpha \rho u\frac{\partial e}{\partial \rho }+i\omega \rho \frac{\partial e}{\partial \rho }+\frac{1}{RePrEc}\frac{\partial \kappa }{\partial \rho }\frac{\partial T}{\partial y}D-i\beta \rho w\frac{\partial e}{\partial \rho }\nonumber \\{} & \quad {} +\frac{1}{RePrEc}\left( \frac{\partial \kappa }{\partial \rho }\frac{\partial ^{2}T}{\partial y^{2}}+\frac{\partial ^{2}\kappa }{\partial \rho ^{2}}\frac{\partial \rho }{\partial y}\frac{\partial T}{\partial y}+\frac{\partial ^{2}\kappa }{\partial \rho \partial T}\left( \frac{\partial T}{\partial y}\right) ^{2}\right) +\frac{1}{Re}\frac{\partial \mu }{\partial \rho }\left( \left( \frac{\partial u}{\partial y}\right) ^{2}+\left( \frac{\partial w}{\partial y}\right) ^{2}\right) \end{aligned}$$

21 $$\begin{aligned}{} & {} {\varvec{\mathsf{{ L}}}}_{5,2}=-i\alpha p+\frac{2\mu }{Re}\frac{\partial u}{\partial y}D,\;{\varvec{\mathsf{{ L}}}}_{5,3}=\frac{2i\alpha \mu }{Re}\frac{\partial u}{\partial y}-pD+i\beta \frac{2\mu }{Re}\frac{\partial w}{\partial y}-\rho \frac{\partial e}{\partial y},\;{\varvec{\mathsf{{ L}}}}_{5,4}=\frac{2\mu }{Re}\frac{\partial w}{\partial y}D-i\beta p\end{aligned}$$

22 $$\begin{aligned}{} {} {\varvec{\mathsf{{ L}}}}_{5,5}&= -\frac{\alpha ^{2}\kappa }{RePrEc}-i\alpha \rho u\frac{\partial e}{\partial T}+i\omega \rho \frac{\partial e}{\partial T}+\frac{1}{RePrEc}\left( \frac{\partial \kappa }{\partial y}+\frac{\partial \kappa }{\partial T}\frac{\partial T}{\partial y}\right) D-i\beta \rho w\frac{\partial e}{\partial T}+\frac{\kappa }{RePrEc}D^{2}-\frac{\kappa \beta ^{2}}{RePrEc}\nonumber \\{} & {} \quad +\frac{1}{RePrEc}\left( \frac{\partial \kappa }{\partial T}\frac{\partial ^{2}T}{\partial y^{2}}+\frac{\partial ^{2}\kappa }{\partial T^{2}}\left( \frac{\partial T}{\partial y}\right) ^{2}+\frac{\partial ^{2}\kappa }{\partial \rho \partial T}\frac{\partial T}{\partial y}\frac{\partial \rho }{\partial y}\right) +\frac{1}{Re}\frac{\partial \mu }{\partial T}\left( \left( \frac{\partial u}{\partial y}\right) ^{2}+\left( \frac{\partial w}{\partial y}\right) ^{2}\right) \end{aligned}$$

Here *D* stands for the differential matrix (using the Chebyshev spectral method) with $D\hat{u}=d\hat{u}/dy$. The perturbations are subject to Dirichlet boundary conditions ($u^{\prime }=v^{\prime }=w^{\prime }=T^{\prime }=0$) at the wall and in the freestream. Compared to the conventional LST framework, the current method has been verified to reproduce the classic results in the ideal-gas regime^38^. As discussed in the previous section, when the fluid operates near its critical point, a perturbation’s growth rate experiences enormous sensitivity to the input of the eigenvalue problem. Therefore, the thermodynamic and transport models are essential to obtaining physically accurate results. A comparison using different equations of state (van der Waals, Redlich–Kwong, Peng–Robinson)^27^ shows no convergence of the result even if the other models are kept the same. Therefore, an accurate look-up table has been used to generate inputs as outlined in the left column of figure 4. The eigenvalue problem (6) is discretized with Chebyshev collocation points and numerically solved with 300 grid points along the wall-normal direction. The results have been tested to be grid-independent.

## Data Availability

The data that support the findings of this study are available from the corresponding author upon reasonable request.
